# Research on Remote GPS Common-View Precise Time Transfer Based on Different Ionosphere Disturbances

**DOI:** 10.3390/s20082290

**Published:** 2020-04-17

**Authors:** Jingkui Zhang, Jingxiang Gao, Baoguo Yu, Chuanzhen Sheng, Xingli Gan

**Affiliations:** 1 School of Environment Science and Spatial Informatics, China University of Mining and Technology, Xuzhou 221116, China; lb18160005@cumt.edu.cn; 2 State Key Laboratory of Satellite Navigation System and Equipment Technology, Shijiazhuang 050081, China; yubg@sina.cn (B.Y.); shengchuanzhen@163.com (C.S.); ganxingli@163.com (X.G.); 3 The 54th Research Institute of China Electronics Technology Group Corporation, Shijiazhuang 050081, China

**Keywords:** propagation path delays, common view, ionosphere disturbance, different ionosphere models, time transfer, time comparison

## Abstract

Propagation path delays are a major error for the remote precise time transfer of common view; these path delays contain the ionosphere and troposphere impact, while the contributions of the ionosphere and the troposphere from common-view satellites to receivers on the ground tend to become uncorrelated when the distance between these receivers increases. In order to select the appropriate ionospheric correction method for common view under different distances between receivers, a detailed test using multi-source data under different ionosphere disturbances are carried out in this paper. Here, we choose three different ionosphere disturbance methods and analyze the advantages and disadvantages of these methods for common-view time transfer and time comparison. At last, we put forward a suitable ionospheric correction method for different distances common view. The RMS shows that the method proposed for 3000 km remote common view can achieve 2.5 ns.

## 1. Introduction

Time and frequency synchronization is an important issue for the cooperative work of manned space flights and spatially distributed carriers, military strike technology, and other areas related to the Internet of Things. How to achieve time transfer and a time comparison between different nodes in a wide range of areas is a particularly tough subject for modern research. At present, there are three main methods commonly used to transmit time and frequency [1]: Two-Way Satellite Time and Frequency Transfer (TWSTFT), Common View (CV) Time Transfer, and two-way optical fiber time transfer technology [1,2,3,4]. The TWSTFT technology needs to rent a precise ephemeris communication satellite, build special transmitting, and receiving signal equipment; these costs are high and cannot be continuous [1,5], currently, only few laboratories have TWSTFT time transfer systems. The two-way optical fiber time transfer technology uses an optical fiber link to transfer the time signal, which has a minimal effect on the environment compared with the TWSTFT and CV technology. However, it requires laying optical fiber, and the cost of fiber regarding time and frequency distribution is too high. Meanwhile, it is not easy to maintain the symmetry of the path delay in very long fiber, so it is not applicable over continental distances [1,5]. The common view is the simplest method for time transfer and time comparison; with the development of the Global Navigation Satellite System (GNSS) navigation system, more satellites can be utilized for common-view time transfer. CV has the advantages of continuity, low cost, and ease of realization; nowadays, it is the primary method in the computation of International Atomic Time (TAI) and UTC [2,3,4,6]. The traditional CV usually utilize pseudo-range measurements, because carrier-phase measurements include nuisance ambiguities, which are difficult to fix, and it needs a long time to achieve the convergence.

The accuracy of the GPS (Global Positioning System) CV method depends on the equality of the orbits and clocks of GPS satellites, propagation path delays between satellite and receivers, and the quality of measurements. In order to reach a better accuracy, effort should be taken to diminish the affection of the error sources [1,2,3,4,6,7,8,9,10,11,12,13]; in the first part, we utilize IGS (International GNSS Service) precise orbit and clock products to eliminate the satellite error.

The propagation path delays contain the ionosphere and troposphere impact, while the troposphere delays are calculated through the Saastamoinen model and random walk estimation model. Ionosphere delays are associated with the distance of the stations: the shorter the distance, the stronger the correlation, and the greater the degree of offset [1,2,3,4]. The present main technologies to weaken ionosphere delays are [14] dual-frequency ionosphere-free combination, the Klobuchar model, the grid model, and the function model, The first method is affected by satellite and receiver instrument deviation and observation noise. The reduced magnitude for the Klobuchar model is relatively little, and it is more suitable for single-frequency users. The grid model depends on the IGS products, which have a certain time delay. Function models are generally suitable for regional ionosphere models with low accuracy. Reference [13,14] explained in detail the theoretical for the Klobuchar model, the dual-frequency combination model, and the Total Electron Content (TEC) map model, and how to apply them. Ionosphere delays is one of the important factors that affect the accuracy of CV, but there is little work that analyzes in detail the impact of ionosphere delays on CV; most of them use empirical models to diminish its influence, or there is no mention of how to deal with the ionosphere delays [15,16,17,18].

The purpose of this paper is to investigate and provide a detailed analysis of the impact of ionosphere disturbance on remote GPS CV. First, we research the law of change about ionosphere disturbance in Section 2.2. Second, we compare the improved effect of the different ionosphere correction models to the real value under different inter-station distances (100 km, 500 km, 1000 km, 3000 km) in Section 3.1. Then, we analyze the GPS CV results of different inter-station distances without ionosphere correction in Section 3.3.1, Next, we research the GPS CV results of different inter-station distances under three different ionosphere correction methods in Section 3.3.2 and Section 3.3.3. Finally, in the final section, the conclusions and future work are summarized.

## 2. Methodology

The mathematical basics for CV time transfer and time comparison, ionosphere impact and different ionosphere delay correction methods, carrier-phase measurements, and smooth pseudo-range measurements are described in detail in this section.

### 2.1. GPS CV Time Comparison Method and Function Model

**Figure 1:**
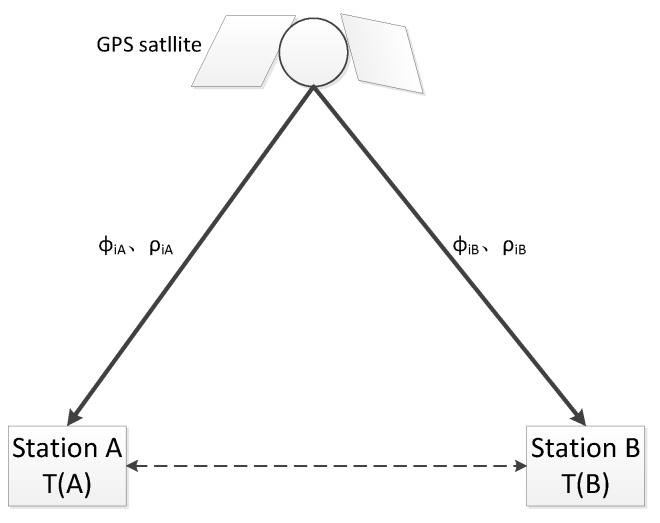
The sketch of Global Positioning System (GPS) Common View (CV).

The CV principle based on GPS is shown in Figure 1 [6]. GPS CV time transfer means two stations observing the very same GPS satellite in the same time to realize the time synchronization of the two stations [6]. If the receivers at stations A and B observe the same GPS satellite ***i*** at the same time, ρ,ϕ are the pseudo-range and carrier-phase measurements. The formula for calculation of the GPS CV result is as follows [3]:

(1) $$c\cdot \delta \;t_{A}=\rho_{kA}-\sqrt{{\left(x^{k}-x_{A}\right)}^{2}+{\left(y^{k}-y_{A}\right)}^{2}+{\left(z^{k}-z_{A}\right)}^{2}}-c\cdot \delta \;t^{k}+ucd_{A}-ucd^{k}-I_{kA}-T_{kA}-\varepsilon_{kA}$$

(2) $$c\cdot \delta \;t_{B}=\rho_{kB}-\sqrt{{\left(x^{k}-x_{B}\right)}^{2}+{\left(y^{k}-y_{B}\right)}^{2}+{\left(z^{k}-z_{B}\right)}^{2}}-c\cdot \delta \;t^{k}+ucd_{B}-ucd^{k}-I_{kB}-T_{kB}-\varepsilon_{kB}$$

where c is the speed of light, $x_{A},y_{A},z_{A}$ are the position of station A, $x_{B},y_{B},z_{B}$ are the position of station B, $x^{k},y^{k},z^{k}$ are the position of satellite ***k***, which can be acquired from the IGS products, $\delta t_{A},\delta t_{B}$ are the signal receive time of station A and station B, $\delta t^{k}$ is the signal send time of satellite ***k***, $ucd_{A},ucd_{B}$ are the code hardware delays of station A and station B, $ucd^{k}$ represents the code hardware delays of satellite ***k***, $T_{kA},T_{kB}$ are the troposphere delays from satellite ***k*** to station A and station B, $I_{kA},I_{kB}$ are the ionosphere delays from satellite ***k*** to station A and station B, and $\varepsilon_{kA},\varepsilon_{kB}$ are the residual errors of station A and station B.

The observation data of each station can be exchanged in real time or afterwards through the network; calculating the difference between Equations (1) and (2), we can get the CV result between station A and station B of satellite ***k***. The following formula is as follows [3,5]:

(3) $$c\cdot \delta \;t_{AB}^{k}=\rho_{kA}-\rho_{kB}-(r_{kA}-r_{kB})-(ucd_{A}-ucd_{B})-(I_{kA}-I_{kB})-(I_{kA}-I_{kB})-(T_{kA}-T_{kB})-(\varepsilon_{kA}-\varepsilon_{kB})$$

where $\delta \;t_{AB}^{k}$ is the CV result between station A and station B of satellite ***k***,

$$r_{kA}=\sqrt{{\left(x^{k}-x_{A}\right)}^{2}+{\left(y^{k}-y_{A}\right)}^{2}+{\left(z^{k}-z_{A}\right)}^{2}},r_{kB}=\sqrt{{\left(x^{k}-x_{B}\right)}^{2}+{\left(y^{k}-y_{B}\right)}^{2}+{\left(z^{k}-z_{B}\right)}^{2}}$$

If the total number of CV satellites between station A and station B are *n*, we take the average of all the satellites as the final clock difference of station A and station B, and the results are

(4) $$c\cdot \delta \;t_{AB}=(\sum_{i=1}^{n}\delta t{\;}_{AB}^{i})/n$$

where $\delta \;t_{AB}$ is the final CV result of station A and station B, and *n* is the number of CV satellites.

According to the above formula, we can conclude that the CV can completely eliminate the error of the satellite clock and most satellite orbit errors. In this article, we use the precise ephemeris provide by IGS for the common-view experiment. The pseudo-range measurements are of meter-level accuracy, and they are susceptible to multipath signals, while the carrier-phase measurements are of millimeter-level accuracy but suffer from an ambiguity-fixed issue, which is difficult to fix and needs a long time to achieve convergence. In this paper, we utilize carrier-phase measurements to smooth pseudo-range measurements to improve the accuracy of pseudo-range measurements. The function model of the double-frequency carrier-phase measurements smoothing pseudo-range measurements is as follows [17]:

(5) $$\begin{array}{l} P_{c}=(P_{1}-g^{2}P_{2})/(1-g^{2})\\ \varphi_{c}=\varphi_{1}/(1-g^{2})-\varphi_{2}/(1-g^{2}) \end{array}$$

where $P_{c},\varphi_{c}$ denotes the pseudo-range and carrier-phase of ionosphere-free combination for each satellite, g = f2/f1, f1, and f2 denote the frequencies of L1 and L2, $P_{1},P_{2}$ are the pseudo-range measurements on the frequencies of L1 and L2. The corresponding original pseudo-range and carrier-phase measurements equations are:

(6) $$\begin{array}{l} \lambda_{c}(\varphi_{c}+n_{c})=\rho +\Delta D_{\varphi_{c}}+\Delta \varepsilon_{\varphi_{c}}\\ P_{c}=\rho +\Delta D_{P_{c}}+\Delta \varepsilon_{P_{c}} \end{array}$$

where $\lambda_{c},n_{c}$ denote the wavelength and integer ambiguity parameters of ionosphere-free combination, $\Delta D_{\varphi_{c}},\Delta D_{p_{c}}$ denote the pseudo-range and carrier-phase multipath effects of ionosphere-free combination, and $\Delta \varepsilon_{\varphi_{c}},\Delta \varepsilon_{p_{c}}$ denote the pseudo-range and carrier-phase residual errors of ionosphere-free combination. We can ignore the multipath effect and residual error with long time observation. Taking the average of epochs, we can attain the ambiguity of an ionosphere-free combination from Equation (6) as follows:

(7) $$\lambda_{c}n_{c}=\sum_{j=1}^{i}\left(P_{cj}-\lambda_{c}\phi_{cj}\right)/i.$$

Then, the smoothed pseudo-range is as follows:

(8) $${\bar{P}}_{ci}=\lambda_{c}\varphi_{ci}+{\langle \lambda_{c}n_{c}\rangle }_{i}$$

(9) $${\langle \lambda_{c}n_{c}\rangle }_{i}={\langle \lambda_{c}n_{c}\rangle }_{i-1}(i-1)/i+(P_{ci}-\lambda_{c}\phi_{ci})/i$$

where ${\langle \rangle }_{i}$ denotes the average of the first *i* epochs, ${\bar{P}}_{ci}$ denotes the smooth pseudo-range of the *i*-th epoch. Utilizing the elimination of carrier-phase measurements with cycle slip to smooth pseudo-range measurements, the final smoothed pseudo-range formula is as follows:

(10) $${\bar{P}}_{ci}=\lambda_{c}\phi_{ci}+{\langle \lambda_{c}n_{c}\rangle }_{i-1}(i-1)/i+(P_{ci}-\lambda_{c}\phi_{ci})/i.$$

### 2.2. Ionospheric Characteristics and Model Analysis

#### 2.2.1. The Relationship Ionospheric Change and the Local Time

To investigate the impact of the ionosphere on CV, we must analyze its features first. This paper utilizes GPS L1 L2 measurements to research the features of the ionosphere, and we can find the relationship between the daily changes of the ionosphere and the local time by numerating the distribution of the vertical total electron content (VTEC) values of one certain station [19,20]. Firstly, we calculate the VTEC value of all satellites that are observed by the receiver of the station. Then, we utilize the distance weighted method (the vertical distance weighted from the ionosphere pierce point to the vertical direction of the station) to numerate the VTEC values of the station [19,20]. The formula is as follows:

(11) $$VTEC_{cezhan}=\sum_{k=1}^{n}\frac{VTEC^{k}}{D_{k}}/\sum_{i=1}^{n}\frac{1}{D_{k}}$$

where $VTEC_{cezhan}$ denotes the VTEC values of the station, $VTEC^{k}$ denotes the VTEC values of the satellite ***k***, and $1/D_{k}$ denotes the weight of satellite ***k***. According to the VTEC values calculation method, we analyze eight days of data from two IGS tracking stations ALBH (48.4° N, 123.5° W) and ANKR (39.9° N, 32.8° W) from 24 January 2016 to 31 January 2016. Considering the limited space in this paper, only the VTEC values calculated on 26 January 2016 are listed separately.

**Figure 2:**
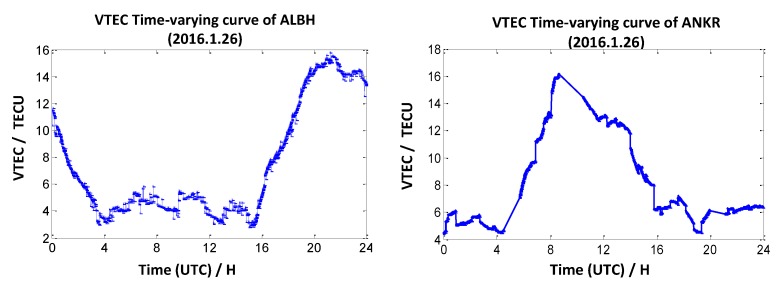
Vertical total electron content (VTEC) time-varying curve of station ALBH and station ANKR.

We draw a conclusion from Figure 2 that the station ALBH reaches the maximum VTEC values at around 21:00 UTC (local time is 13:00), while the VTEC values reach the minimum at around 15:00 UTC (local time is 6:00), and the station ANKR reaches a maximum VTEC value at around UTC 9:00 (local time is 12:00), and it reaches its minimum VTEC value at around 4:00 UTC (local time is 7:00). It is obvious that the change of VTEC values is consistent with the law of ionosphere variation (it peaks at 11:00–13:00 local time and reaches the minimum value at 6:00–7:00 local time).

#### 2.2.2. Feature of Three Different Ionosphere Methods

**Figure 3:**
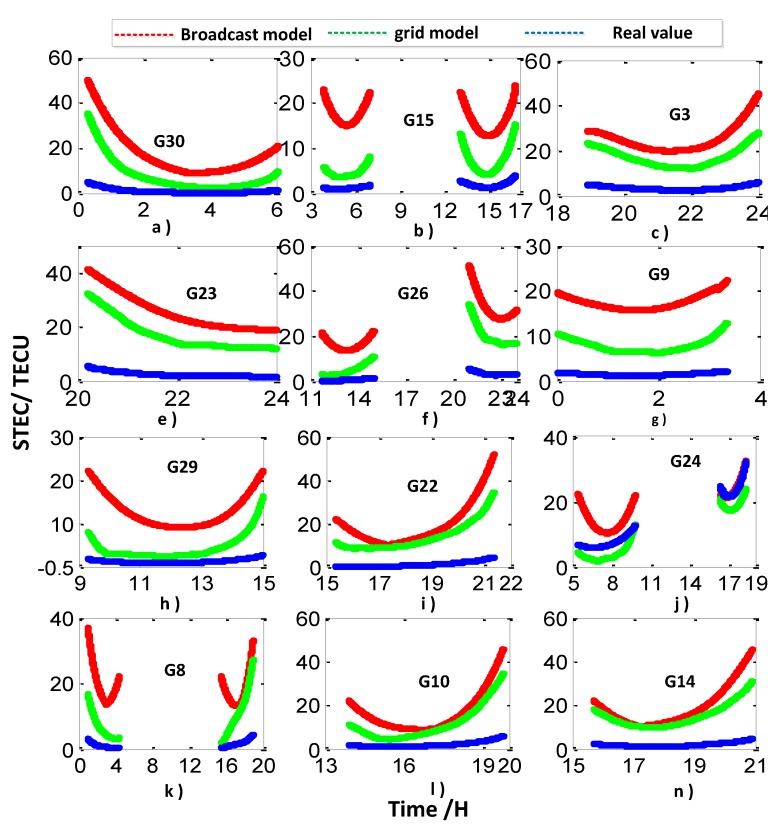
Three different ionosphere methods of correction trace over time for the ALBH station.

In this section, we utilize three different ionosphere methods to research their impact on CV; the three different ionosphere methods are the broadcast ionosphere model (broadcast model for short), the grid ionosphere model (grid model for short), and the ionosphere-free combination methods. The changing curves of the three different ionosphere methods at the IGS tracking station ALBH (48.4° N, 123.5° W) on 26 January 2016 are used to analyze the characteristics of the three ionosphere methods. We chose 12 satellites with tracking time greater than two hours of the day at ALBH station for researching; the time variation results under three different ionosphere methods are addressed in Figure 3.

The ionosphere delay values calculated by geometry-free combination are taken as the real values. From the above graph, we can conclude that the ionosphere delay values calculated by the grid model are closer to the real values than those of the broadcast model. At present, the broadcast model has been proven to be a convenient, reliable, and practical method for ionosphere correction; however, this model can amend approximately 60%–70% compared to the real values in the middle latitudes areas. In the high and low latitudes areas where the ionosphere are more active, the model may be worse [21,22,23,24].

The grid model utilizes the post products provided by IGS to eliminate the ionosphere impact. This model is related to the distribution density of monitoring stations in specific areas; the correction can achieve good accuracy when the distribution of monitoring stations is dense, but the effect is poor when the distribution of monitoring stations is sparse [24].

The main impact of the ionosphere is the first-order delays, which can be eliminated by utilizing ionosphere-free combination. It is observable that the residual higher-order delays are less than 0.5 ns for the CV time transfer; hence, they are usually being ignored. Meanwhile, the ionosphere-free combination observable can amplify the observable noise [22]. When the distance of the inter-station is short, the accuracy of time comparison will obviously decline due to the noise [22].

## 3. Implementations and Evaluation

### 3.1. The Delays of Three Different Ionospheric Methods under Different Inter-Station Distances

**Figure 4:**
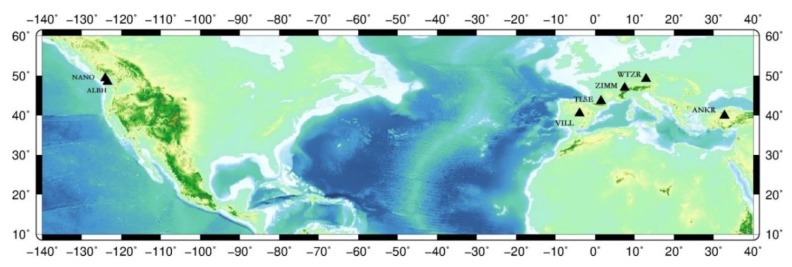
Geographical distribution of the International GNSS Service (IGS) stations.

In order to verify the impact of different ionosphere methods on an inter-station time comparison, in this paper, the original observation data of seven IGS tracking stations (NANO, ALBH, TLSE, ANKR, WTZR, ZIMM, and VILL) on 26 January 2016 are selected for analysis. Four pairs (NANO-ALBH, TLSE-ZIMM, TLSE-WTZR, VILL-ANKR) are chosen from the seven stations, the interval of the observation data is 30 s, and the distance range of these groups is 100 to 3000 km. The ionosphere delay values of different methods between the four groups of stations were calculated respectively, the ionosphere delays value calculated by geometry-free combination is taken as the real value. The information of the CV stations is shown in the Figure 4 and Table 1.

**Figure 5:**
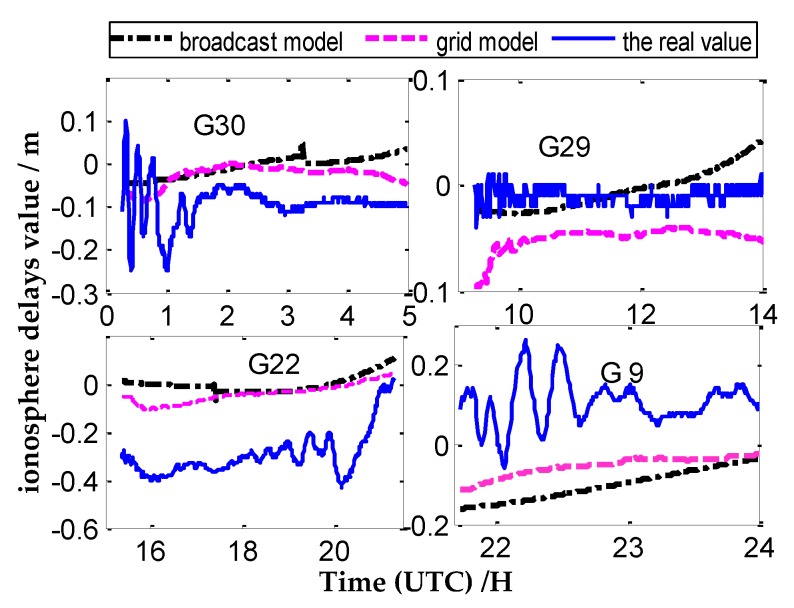
The ionosphere delay values under three ionosphere methods of NANO and ALBH.

**Figure 6:**
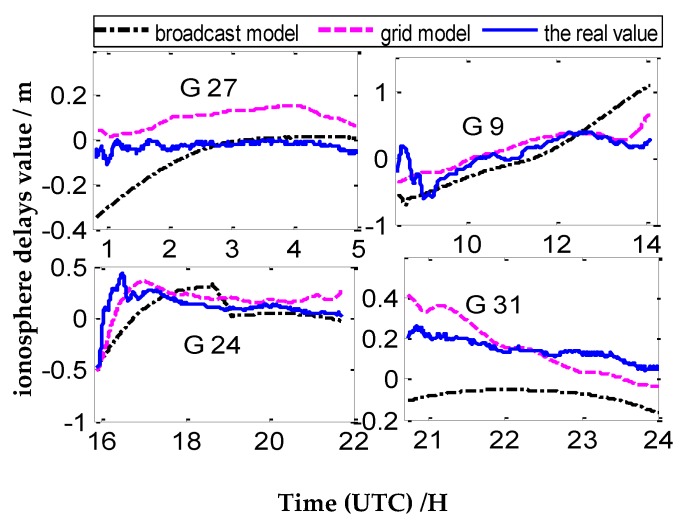
The ionosphere delay values under three ionosphere methods of TLSE and ZIMM.

**Figure 7:**
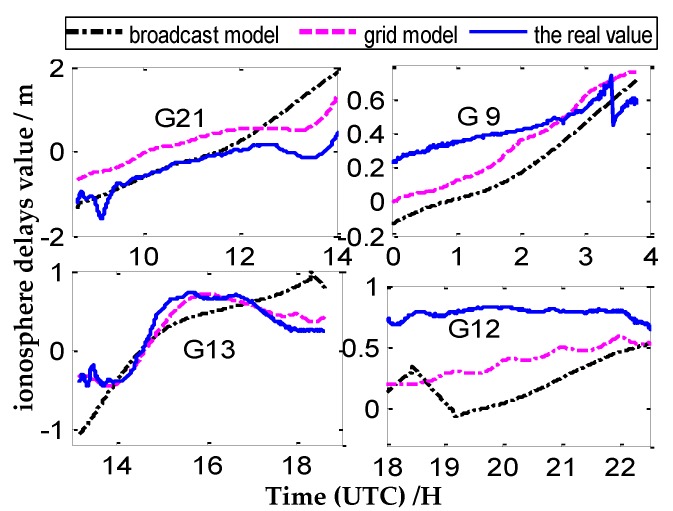
The ionosphere delay values under three ionosphere methods of TLSE and WTZR.

**Figure 8:**
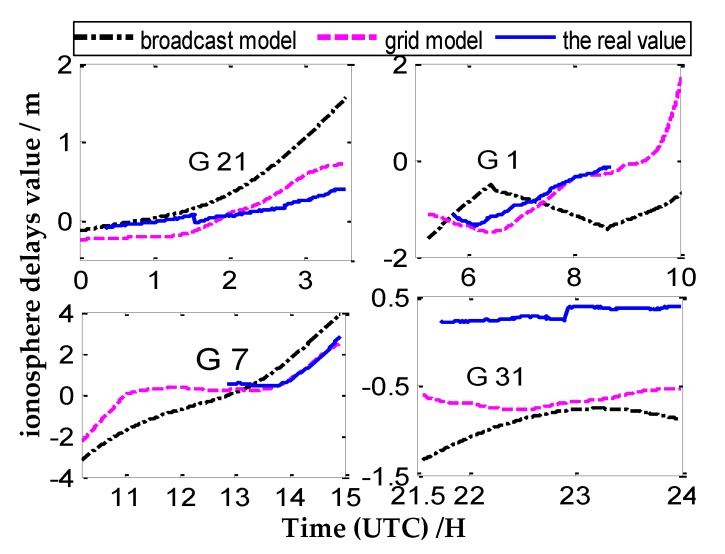
The ionosphere delay values under three ionosphere methods of VILL and ANKR.

Four CV satellites at different times are selected from the four pairs CV stations to represent the variation of ionosphere delays of one day, The number of CV satellites observed between close stations are more, and the tracking time of common satellites is relatively long (as shown in Figure 5). Meanwhile, the number of CV satellites is less between remote stations, and the tracking time of common satellites is relatively short (as shown in Figure 5, Figure 6, Figure 7 and Figure 8).

According to Figure 5, Figure 6, Figure 7 and Figure 8, we draw a preliminary conclusion: among NANO and ALBH, TLSE and WTZR, and TLSE and ZIMM, compared with the real values of ionosphere delays, the difference calculated by the broadcast model is between 0.1 and 0.7 m, and the results of the grid model are better than those of the broadcast model. From satellite 31 of VILL and ANKR, we can see that the difference between the broadcast model values and real values is often more than 1 m, and the maximum differ is 2 m, while the difference between the grid model and real value is less than 1 m. From the remaining three satellites of VILL and ANKR, the difference between the grid model and real value is basically the same. As a whole, the results of the ionosphere delays calculated by the grid model are similar to those of the real value, while the trends and results of the broadcast model are worse than those of the grid model.

### 3.2. Process Strategies and Models

In order to verify the characteristics of ionosphere impact on time transfer and time comparison, we utilize the data provided by IGS for experimental analysis. First, we analyze the results and accuracy of remote GPS CV without any ionosphere correct. Then, we analyze the results and accuracy of remote GPS CV calculating by three different ionosphere methods: a broadcast model, a grid model, and an ionosphere-free combination method. Then, we take the inter-station clock difference provided by IGS as the true value. The seven-day observed and precise ephemeris data from 24 January 2016 to January 30 2016 of the four groups CV stations mentioned above are utilized, and every day’s data is processed separately.

The parameter site strategy in this paper is shown in Table 2. The troposphere correction utilizes the Saastamoinen model to correct its dry component, while the remainder of the wet component is estimated by random walk estimation, and we utilize the GMF mapping function to map the zenith delay to the slant observation path [25,26]. Furthermore, the solid Earth tides, relativistic effect, ocean loading, Earth rotation, and satellite antenna phase center deviation are corrected with the corresponding models, while Different Code Bias (DCB) correction utilizes the IGS products [24].

### 3.3. Experiment and Analysis

#### 3.3.1. Experiment without Ionosphere Correction

**Figure 9:**
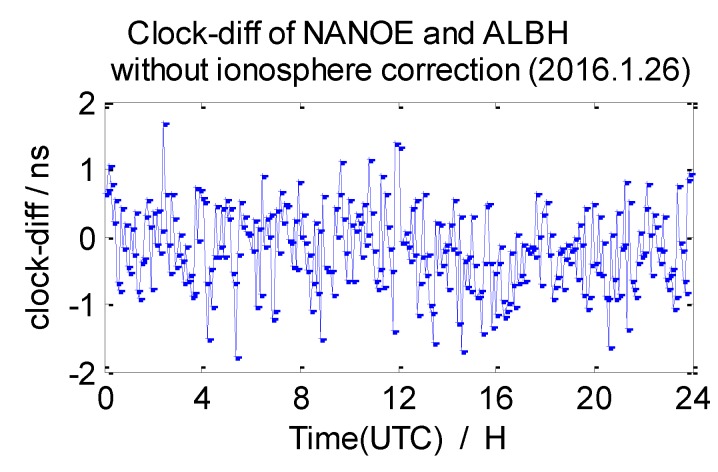
Clock difference between NANO and ALBH (without ionosphere correction).

**Figure 10:**
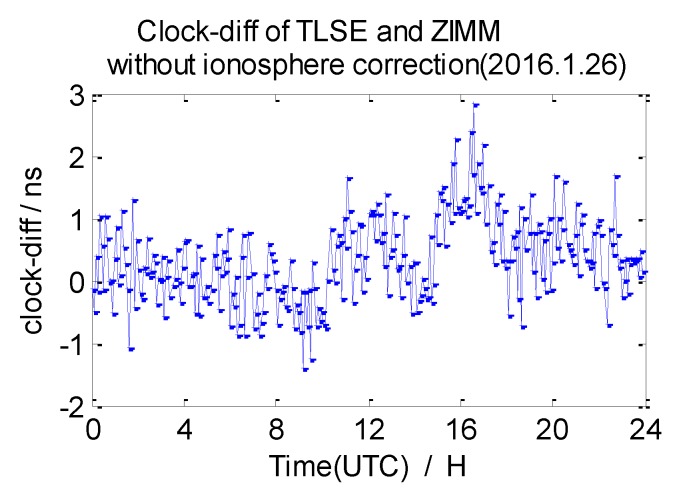
Clock difference between TLSE and ZIMM (without ionosphere correction).

**Figure 11:**
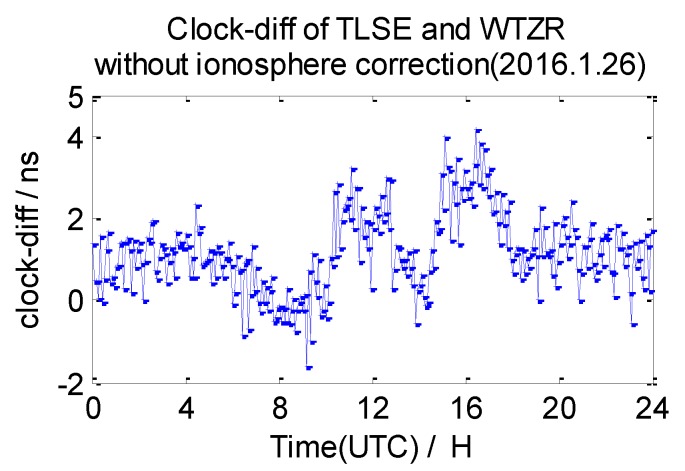
Clock difference between TLSE and WTZR (without ionosphere correction).

**Figure 12:**
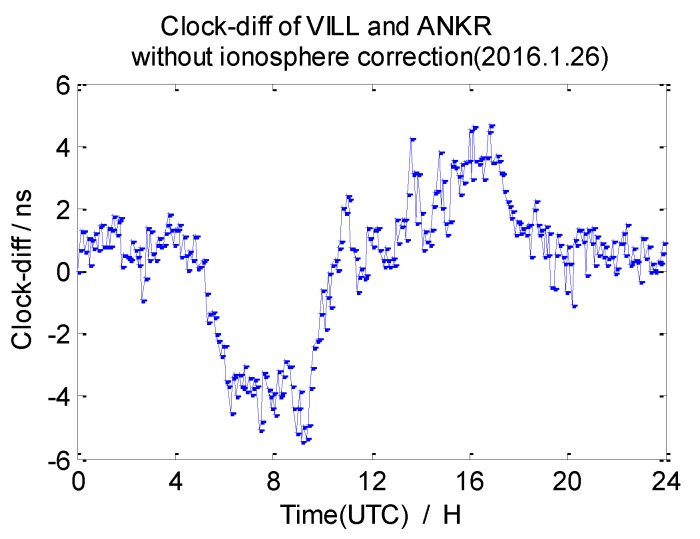
Clock difference between VILL and ANKR (without ionosphere correction).

The average 7-day clock difference results of CV without ionosphere correction are calculated according to the formula mentioned in Section 2.1, the Root Mean Square (RMS) of the clock differences is shown in Table 3. Here, we list the clock difference result chart of four groups from 26 January 2016 in Figure 9, Figure 10, Figure 11 and Figure 12.

From the data in Table 3, we draw a preliminary conclusion: the correlation of ionospheres becomes weaker when the distance increases, the clock difference results of CV inter-station are affected by the weaker correlation; the distance between NANO and ALBH is 109 km, and the average RMS of the clock difference of 7 days is 0.74 ns; the distance between TLSE and ZIMM is 595 km, and the average RMS of the clock difference of 7 days is 1.07 ns; the distance between TLSE and WTZR is 1071 km, and the average RMS of the clock difference of 7 days is 1.98 ns, while the distance between VILL and ANKR is 3000 km, and the average RMS of the clock difference of 7 days is 3.17 ns. It is obvious that the wave range of the clock difference is −2 ns to 2 ns between NANO and ALBH on 26 January 2016, when the distance reaches 1071 km, the wave range −2 ns to 4 ns which shows little change, but the wave range achieves −6 ns to 6 ns when the distance reaches 3000 km on the same day. Meanwhile, the variation is consistent with the law of ionosphere variation when the distance between these stations increases. Therefore, the ionosphere delay impact must be considered in remote high precision GPS CV for nanosecond magnitudes accuracy.

#### 3.3.2. Experiment with Three Different Ionosphere Correction Methods

**Figure 13:**
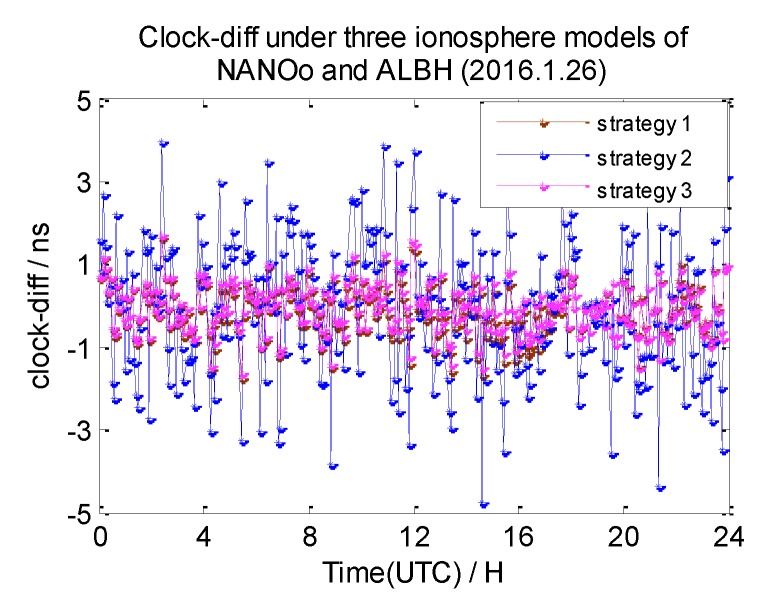
The clock difference under three ionosphere methods of NANO and ALBH.

**Figure 14:**
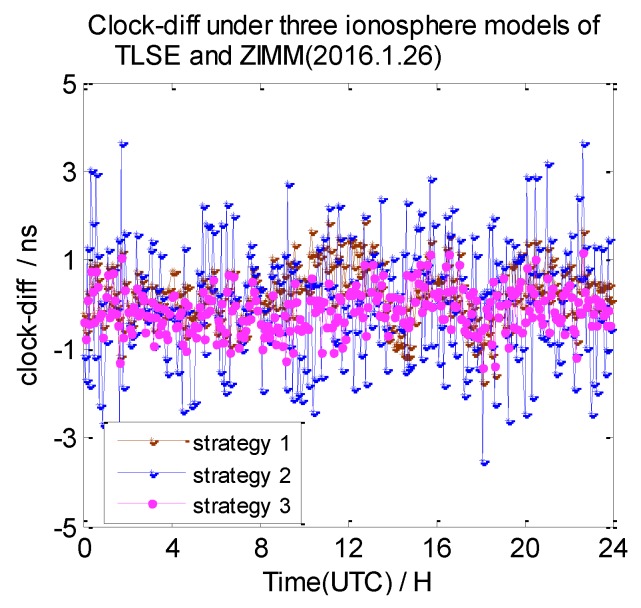
The clock difference under three ionosphere methods of TLSE and ZIMM.

**Figure 15:**
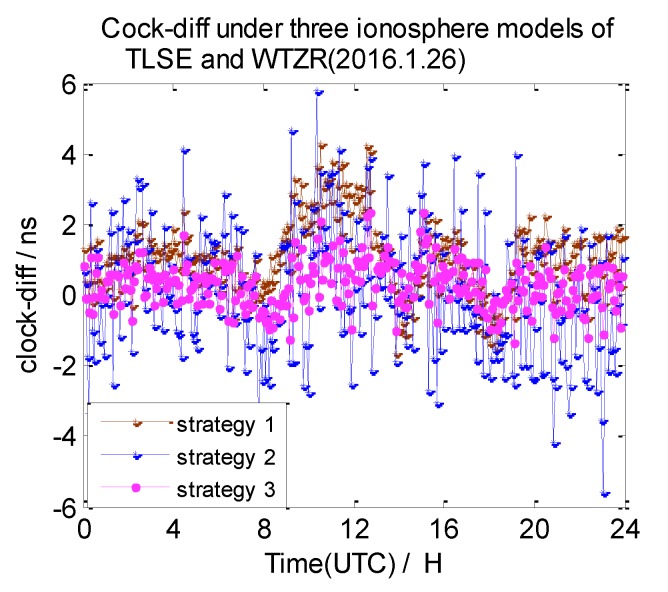
The clock difference under three ionosphere methods of TLSE and WTZR.

**Figure 16:**
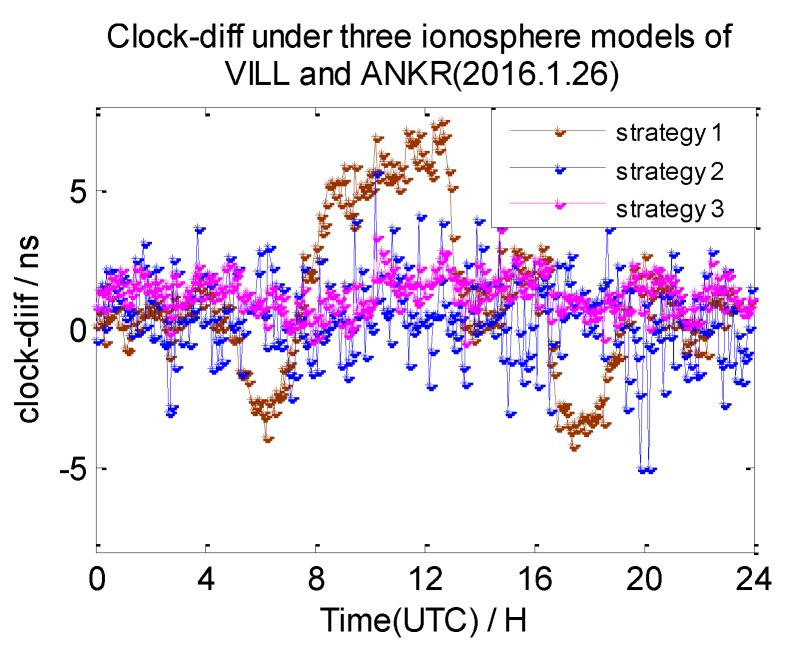
The clock difference under three ionosphere methods of VILL and ANKR.

In this section, the 7-day clock difference results of CV under three different ionosphere correction methods are calculated. The RMS of the clock differences are shown in Table 4. Here, we also list the clock difference result charts of four groups on 26 January 2016 in Figure 13, Figure 14, Figure 15 and Figure 16. We define the broadcast model as strategy 1, the ionosphere-free combination method as strategy 2, and the grid model as strategy 3, 

From these graphs and the data in the table above, we can draw a conclusion: due to the ionosphere-free combination method amplifying the observed noise, strategy 2 has the worst clock results on NANO and ALBH, TLSE and ZIMM, and TLSE and WTZR, whose inter-station distances are less than 1000 km. As the inter-station distance increases, the ionosphere correlation gradually becomes weaker, and the impact of the amplified noise also gradually becomes smaller; when the distance is up to 3000 km, the clock results of strategy 2 are better than those of strategy 1 and strategy 3.

Compared to the clock difference results without ionosphere correction, the clock difference result of the four groups used in strategy 1 are improved by 16%, 36%, 29%, and 18%, while the clock difference result of the four groups used in strategy 3 are improved by 22%, 36%, 65%, and 55%. The clock difference correction effect of strategy 1 is relatively small and becomes worse with the increase of distance; when the distance reached 3000 km, the clock difference result only improved 18% compared to the strategy without ionosphere correction. It is obvious that strategy 1 cannot satisfy the demand of remote GPS. Strategy 3 works best, but it relies on the products provided by IGS.

#### 3.3.3. Double-Frequency Carrier-Phase Measurements Smooth Pseudo-Range Measurements Method

**Figure 17:**
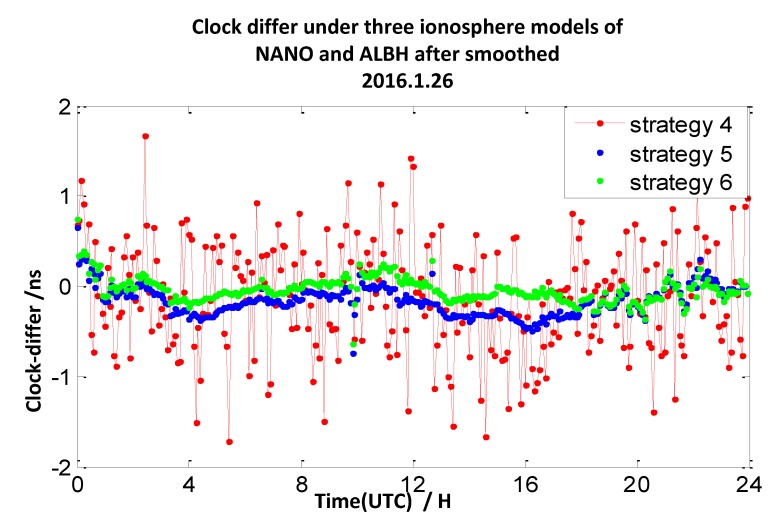
The clock difference under three ionosphere models of NANO and ALBH after being smoothed.

**Figure 18:**
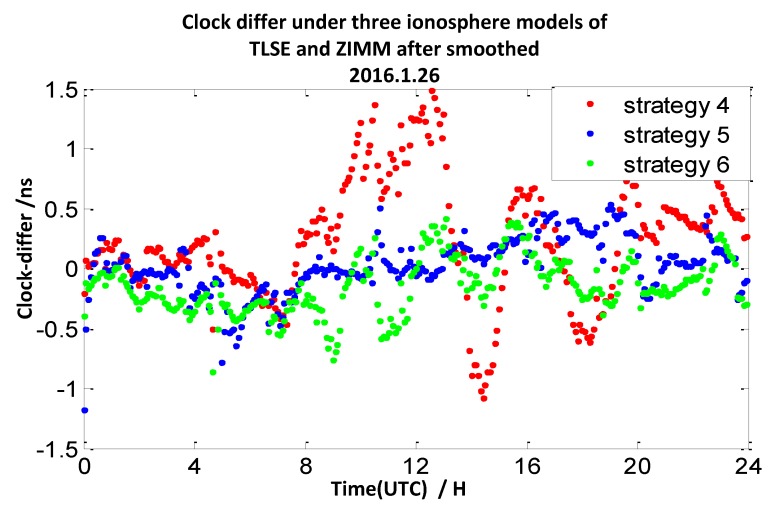
The clock difference under three ionosphere models of TLSE and ZIMM after being smoothed.

**Figure 19:**
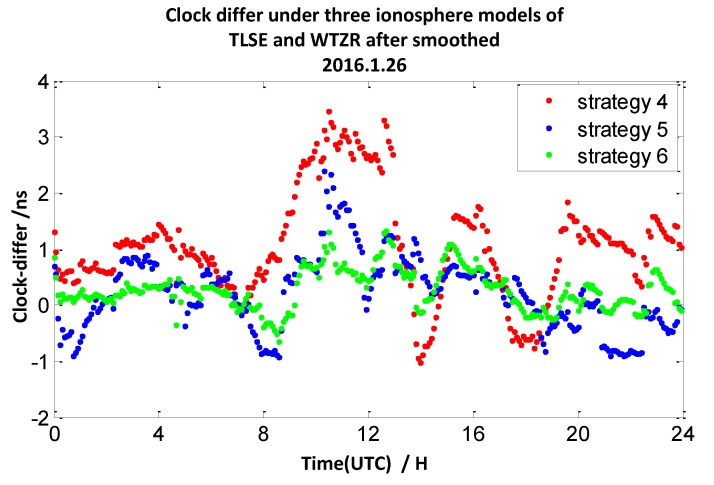
The clock difference under three ionosphere models of TLSE and WTZR after being smoothed.

**Figure 20:**
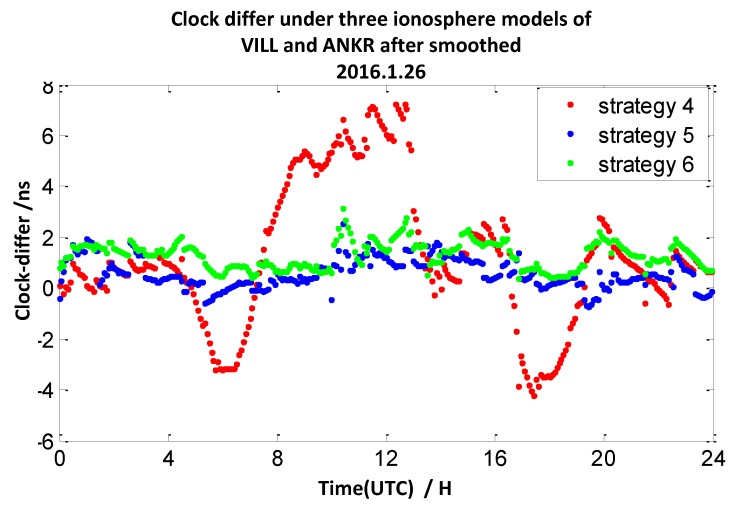
The clock difference under three ionosphere models of VILL and ANKR after being smoothed.

The clock difference results calculated by strategy 2 mentioned above utilize double-frequency pseudo-range measurements. In this section, we utilize the ionosphere-free combination of L1 and L2 measurements to smooth the P1 and P2 measurements to calculate the clock difference results of the four pairs of stations (mentioned in Section 2.1). Here, the interval of the observation data is 30 s, and the interval of the smooth is 10 min. We define the broadcast model with carrier-phase measurements and smooth pseudo-range measurements as strategy 4, the ionosphere-free combination method with carrier-phase measurements and smooth pseudo-range measurements as strategy 5, and the grid model with carrier-phase measurements and smooth pseudo-range measurements as strategy 6. The 7-day clock difference RMS results are shown in Table 5, and the clock difference result chart of four groups on 26 January 2016 are illustrated in Figure 17, Figure 18, Figure 19 and Figure 20.

From these graph and the data in the table above, the results indicate that compared with strategy 1, the RMS results of strategy 4 under TLSE-WTZR and VILL-ANKR get worse, and the improvement under NANO-ALBH and TLSE-ZIMM is very little. Compared with strategy 2, when utilizing strategy 5, the clock difference results are significantly improved, and the improvement percentages are 74%, 77%, 57%, and 43%. Compared to strategy 3, when utilizing strategy 6, the clock difference results are significantly improved, and the improvement percentages are 76%, 62%, 25%, and 0.7%.

We can conclude that the accuracy has little or no improvement when utilizing the broadcast model with carrier-phase measurements and smooth pseudo-range measurements. There is an obvious improvement when utilizing an ionosphere-free combination method with carrier-phase measurements and smooth pseudo-range measurements: the range of improvement under the four pairs of CV stations is from 43% to 77%. Under the grid model with carrier-phase measurements and smooth pseudo-range measurements, the improvement effects are obvious when the distance between stations is less than 500 km, while it gradually decreases when the distance is more than 1000 km, and the improvement is only 0.7% under the pair of VILL-ANKR, where the distance is 3000 km. Under long distance conditions, the methods in strategy 5 are better than those of the other models, when the distances are less than 1000 km, strategy 6 is the suitable method, but it relies on IGS products.

## 4. Conclusions

The ionosphere has a fatal impact on the remote GPS CV precise time transfer. In this paper, we research the impact of three different ionosphere correction methods and compare their impact on the CV time transfer and time comparison, and we analyze the advantages and disadvantages of the different ionosphere methods under different distances. Utilizing one week of GPS data from seven globally distributed IGS tracking stations in the year of 2016 to carry out our CV experiment, we choose 4 pairs with distance ranges from 100 to 3000 km from the seven stations. Finally, we come to the conclusion after detailed analysis that when the distance between stations is less than 1000 km, the grid model with carrier-phase measurements and smooth pseudo-range measurements is the best method, in which the RMS of the clock error is 0.52 ns. When the distance reached 3000 km, the ionosphere-free combination method with carrier-phase measurements and smooth pseudo-range measurements is the best method; the time synchronization accuracy of common view can reach 2.5 ns, and the RMS of the clock error is 0.87 ns. Thus, it can completely satisfy the demand of nanosecond magnitude precision and remote GPS CV.

The testing data sets utilized in this thesis are from the permanent stations, which have very good observation environments, but the multipath effect is not considered throughout this thesis. However, these prerequisites might be hard to be satisfied in real-time practice. Therefore, the actual performances of the observations at the non-permanent stations and the carrier phase pseudo-range observations would be considered in further research.

## Figures and Tables

**Table 1:** The information of Common View (CV) stations.

Station A	Station B	Distance/km
NANO	ALBH	109
TLSE	ZIMM	595
TLSE	WTZR	1071
VILL	ANKR	3000

**Table 2:** The parameters selection methods.

	Parameter	Process Methods
Observations	Type of data	P1,P2
	Cut-off elevation	7°
Error process	Carrier-phase wind-up	model correction
	Solid earth tides DCB	model correction model correction
	Earth rotation parameters	model correction
	Weight	elevation angle weight
	Antenna model	IGS08.ATX
	Troposphere	Saastamoinen model and random walk estimation

**Table 3:** Clock differ RMS of 7 days without ionosphere correction between stations.

	NANO-ALBH	TLSE-ZIMM	TLSE-WTZR	VILL-ANKR
RMS (ns)	0.74	1.07	1.98	3.17

**Table 4:** The clock difference RMS of 7 days under three ionosphere models.

	NANO-ALBH	TLSE-ZIMM	TLSE-WTZR	VILL-ANKR
Strategy 1 RMS (ns)	0.62	0.68	1.41	2.59
Strategy 2 RMS (ns)	1.59	1.34	1.69	1.57
Strategy 3 RMS (ns)	0.58	0.68	0.69	1.42

**Table 5:** The clock difference RMS of 7 days before and after phase smoothed.

	NANO-ALBH	TLSE-ZIMM	TLSE-WTZR	VILL-ANKR
Strategy 4 RMS (ns)	0.61	0.57	1.49	3.01
Strategy 5 RMS (ns)	0.42	0.31	0.72	0.89
Strategy 6 RMS (ns)	0.14	0.26	0.52	1.41
